# E2F1 activation is responsible for pituitary adenomas induced by HMGA2 gene overexpression

**DOI:** 10.1186/1747-1028-1-17

**Published:** 2006-08-17

**Authors:** Monica Fedele, Giovanna Maria Pierantoni, Rosa Visone, Alfredo Fusco

**Affiliations:** 1 Istituto di Endocrinologia e Oncologia Sperimentale del CNR e Dipartimento di Biologia e Patologia Cellulare e Molecolare, Università di Napoli "Federico II", via S. Pansini, 5 80131 Napoli, Italy; 2 NOGEC (Naples Oncogenomic Center) – CEINGE Biotecnologie Avanzate & SEMM – European School of Molecular Medicine – Naples Site, via Comunale Margherita 482, Naples, Italy

## Abstract

The High Mobility Group protein HMGA2 is a nuclear architectural factor that plays a critical role in a wide range of biological processes including regulation of gene expression, embryogenesis and neoplastic transformation. Several studies are trying to identify the mechanisms by which HMGA2 protein is involved in each of these activities, and only recently some new significant insights are emerging from the study of transgenic and knock-out mice. Overexpression of *HMGA2 *gene leads to the onset of prolactin and GH-hormone induced pituitary adenomas in mice, suggesting a critical role of this protein in pituitary tumorigenesis. This was also confirmed in the human pathology by the finding that HMGA2 amplification and/or overexpression is present in human prolactinomas. This review focuses on recent data that explain the mechanism by which HMGA2 induces the development of pituitary adenomas in mice. This mechanism entails the activation of the E2F1 protein by the HMGA2-mediated displacement of HDAC1 from pRB protein.

## Background

Pituitary tumors constitute 10% of intracranial neoplasms, and are mostly benign with slow growth [1]. Most pituitary neoplasms secrete hormone gene products, leading to disturbed endocrine functions. Prolactinomas account for the most common type of pituitary adenomas [1,2], while about one-third of pituitary adenomas are not associated with clinical hypersecretory syndromes, but with symptoms of an intracranial mass that leads to headaches, hypopituitarism or visual-field disturbances, which are classified as non-functioning pituitary adenomas (NFPAs). The genesis of pituitary tumors is still mainly unknown, but the actual model supposes that genetic alterations represent the initializing event that transforms pituitary cells, and that hypothalamic hormones and other local growth factors may play an important role in promoting the growth of already transformed cells. However, the classical gene alterations involved in cell transformation, such as *ras*, *BRAF*, *Rb*, do not appear to be responsible for the onset of pituitary adenomas [3]. Only up to 40% of sporadic human GH-secreting adenomas have missense mutations of the Gsα gene [4], and many functional adenomas present the overexpression of a recently discovered powerful transforming gene, *PTTG*, which is able to exert strong transforming effects both *in vitro *and *in vivo *[5].

**Figure 1:**
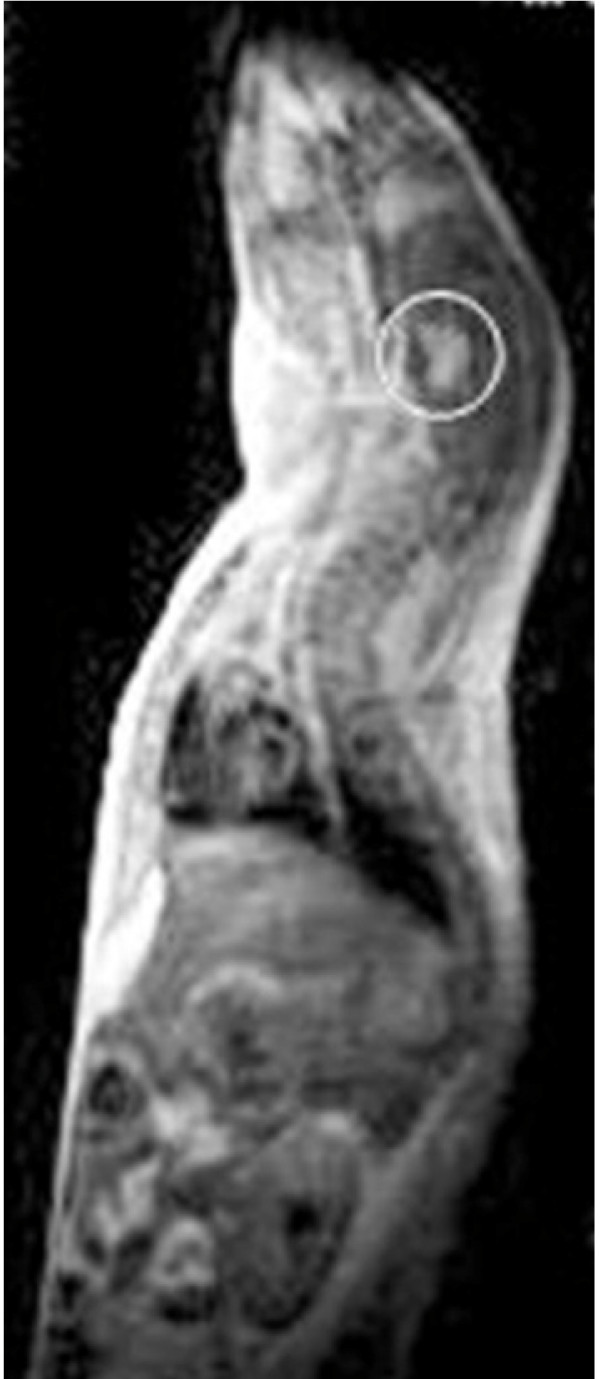
Magnetic Resonance Image of a *HMGA2 *transgenic mouse showing a pituitary adenoma (indicated in circle).

Recently, our group suggested a critical role for high-mobility group A2 (HMGA2) gene in pituitary oncogenesis. In fact, transgenic mice expressing high levels of the *HMGA2 *gene develop pituitary adenomas secreting prolactin and growth hormone [6], (Figure 1).

The HMGA2 protein belongs to the HMGA family. The HMGA protein family members are non-histones, small, nuclear proteins, that bind the minor groove of AT-rich DNA sequences through their "AT-hook" domains localised in the N-terminal region of the proteins [7]. These proteins play key roles in chromatine architecture and gene control by serving as generalized chromatin effectors, either enhancing or suppressing the ability of more usual transcriptional factors to act in transcriptional regulation [8].

HMGA2 expression is restricted during embryogenesis, whereas it is absent or very low in normal adult tissues [9,10]. Induction of *HMGA2 *gene expression occurs in several human malignant neoplasias, including thyroid [11,12], pancreas [13], breast [14], and colorectum [15-17], and seems to play a critical role in cell transformation, since the block of its synthesis prevents rat thyroid transformation by murine transforming retroviruses [18]. Conversely, rearrangements of the *HMGA2 *gene are frequently detected in human benign tumors of mesenchymal origin [19]. Consistent with the onset of pituitary adenomas in *HMGA2*-transgenic mice, we have found the induction of HMGA2 expression in human prolactinomas in association with amplification and/or rearrangement of the gene [20], and, recently, we have shown that also the majority of NFPAs express HMGA2, but, in these cases, it is not associated to over-representation of the *HMGA2 *region [21].

## HMGA2 binds to pRB and inhibits its function

The Retinoblastoma protein, pRB, has been suggested to be a key protein in the pituitary tumorigenesis because of the pituitary tumors developed by *RB+/- *mice [22], but no RB mutations, apart from few high aggressive pituitary carcinomas [23], have been so far reported in human pituitary pathology [24]. However, methylation of the *RB *gene-promoter region at a CpG island, resulting in loss of protein expression, has been described in human pituitary tumor cells [25], suggesting that pRB is indeed critical in human pituitary tumorigenesis.

pRB controls cell cycle progression through its interaction with the E2F family of transcription factors [26,27], whose activity is crucial for the expression of several genes required to enter the S phase of the cell cycle [28,29]. The transcriptional activity of E2F1 is repressed in non-proliferating cells by its interaction with pRB that masks the activation domain of E2F1, and prevents it to contact the general transcription machinery [30]. Conversely, in proliferating cells, pRB is phosphorylated at multiple sites by cyclin-dependent kinases [31,32], resulting in the release of E2F1 and, consequently, transcriptional activation of its target genes [33]. More recently, a new mechanism of pRB-mediated E2F1 repression has been suggested in addition to this one. It is an active repression that pRB exerts on E2F1-mediated transcription by recruiting class I histone deacetylase proteins (HDAC1) to the E2F1-sites. The HDACs repress transcription by removing acetyl groups from the histones, thereby facilitating the condensation of nucleosomes into chromatin and therefore blocking access to transcription factors [34].

Based on the striking mirror similarities between the phenotypes of pRB [22,35] and HMGA2 [36,37] animal models, our group has recently investigated a potential functional interaction between HMGA2 and the Retinoblastoma protein [38]. By co-immunoprecipitating HMGA2 and pRB in pituitary adenomas developed by *HMGA2 *mice, we demonstrated the interaction between the two proteins occurring in the tumor. This interaction was then repeated and confirmed *in vitro *with recombinant proteins, finding that one of the pRB domains involved in the interaction is the A/B pocket [30], the same domain that is also involved in the interaction with E2F1, HDAC1 and viral oncoproteins such as those produced by the E1A adenovirus [39,40]. This was very interesting because it suggested that HMGA2, similarly to the viral oncoproteins, could inhibit pRB function by displacing E2F1 and HDAC1 from pRB. By transfection, luciferase and colony assays, we could establish that the overexpression of HMGA2 antagonizes the activity of pRB. In fact it blocks the pRB-dependent inhibition of both E2F1 target gene transcription and cell proliferation. Interestingly, this positive role of HMGA2 on cell proliferation is due to the interaction with pRB, opening a new class of cell cycle related proteins: "the suppressors of the cell cycle inhibitors". As described above, HMGA2 is considered a *bona fide *oncogene because it induces both neoplastic transformation of cultured rat fibroblasts [41] and tumors in transgenic mice [6]. Interestingly, we found that the interaction between HMGA2 and pRB is crucial for the transforming activity of HMGA2 protein. In fact, in a focus assay on rat fibroblasts, HMGA2 mutants unable to bind pRB lost the capacity of the wild-type gene to transform cells. These results suggest that the binding between HMGA2 and pRB may be generally involved in HMGA2-mediated cell transformation.

## HMGA2 displaces HDAC1 from E2F1 target promoters and causes acetylation of both histones and E2F1 protein

**Figure 2:**
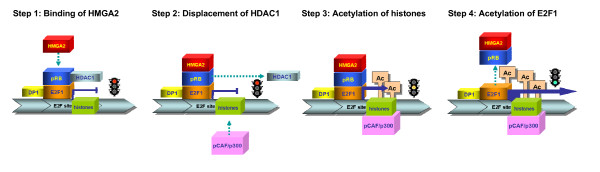
**Schematic model of E2F1 activation by HMGA2**. Following HMGA2 overexpression, transcription through E2F1 sites switches from repression to activation through four steps: 1- HMGA2 binds to pRB, which is complexed with E2F1 and HDAC1 to form the active repression; 2- the interaction between HMGA2 and pRB displaces HDAC1; 3- in the absence of HDAC1, the histone acetylase enzymes are recruited and, by acetylating histones, relieve transcriptional repression; 4- histone acetylases also acetylate E2F1 causing the stabilization of its "free" active form.

Using competitions with recombinant proteins and Chromatin Immonoprecipitation (ChIP) experiments, we demonstrated that following the binding of HMGA2 to pRB (Figure 2, step1), HDAC1 is displaced from the E2F1-target promoters (Figure 2, step 2) where it was recruited by pRB [34]. Consistently, HDAC1 activity associated to pRB is lower in cells and pituitary adenomas overexpressing HMGA2 than in mock-transfected cells and normal pituitary, respectively [38]. Histone acetyl transferases and histone deacetylases acetylate and deacetylate core histone tails that protrude from the nucleosome. Histone acetylation is thought to weaken the interaction between histone N-terminal tails and DNA, thus opening up the chromatin and increasing accessibility for activating transcription factors [42,43]. Therefore, the displacement of HDAC1 from pRB results in the recruitment of histone acetyl transferase to the E2F1-target promoters and acetylation of both histones and other proteins, including E2F1. This was convincingly demonstrated by ChIP experiments using antibodies against acetylated histone H3 and E2F1 [38]. The acetylation of both histones and E2F1 protein increase about two-fold the E2F1 transcriptional activity. In fact, as above described, the acetylation of histones opens up the chromatin and facilitates gene transcription (Figure 2, Step 3). Moreover, acetylation of E2F1 augments its DNA binding and stabilizes the protein in its "free" active form [44] (Figure 2, Step 4). Thus, as a consequence of the E2F1 acetylation, HMGA2 can indirectly also cause the displacement of E2F1 from pRB as it was observed by ChIP and re-ChIP experiments on the cyclin E1 promoter [38].

## Suppression of pituitary tumorigenesis in HMGA2 transgenic mice lacking E2F1

Does the afore-reported HMGA2-dependent molecular events result in enhanced E2F1-dependent gene transcription in pituitary adenomas? The affirmative answer comes once again from the study of the *HMGA2 *transgenic mice. In fact, pituitary adenomas excised from these mice were used in EMSA assays to analyze the E2F1-DNA binding in pituitary tumours compared to normal pituitary glands from wild-type mice [38]. The data obtained showed a drastic increase of the "free" active form of the E2F/DNA complex. Moreover, by RT-PCR and ChIPs on tissues, expression of E2F-target genes, such as *CDC1 *and *TK1*, was shown to be enhanced, and E2F1 to be more acetylated in adenomas compared to normal glands (unpublished data). This suggests that E2F1 activity is a critical event in pituitary tumorigenesis of HMGA2 mice.

To address this hypothesis, we crossed *HMGA2 *transgenic mice with *E2F1 *knock-out mice to generate double mutants [38]. With our big satisfaction, the hypophysis of these mice was only rarely and however minimally interested to the adenomatous phenotype. In fact, the adenoma was diagnosed in only 25% of double mutant mice in respect to *HMGA2 *transgenic mice which all developed pituitary tumors. Moreover the tumours of the mice lacking *E2F1 *were smaller and slower growing than those developed by the *HMGA2 *mice. Interestingly, even in pituitary adenomas developed by *HMGA2 *mice lacking E2F1 the interaction between HMGA2 and pRB was present, however, the E2F "free" DNA binding activity did not show any significant increase compared to control wild-type glands. Conversely, an increase in E2F "free" DNA binding was always observed in pituitaries from single mutant *HMGA2 *mice even before the appearance of the pituitary tumour. Thus, even though HMGA2 is still able to bind pRB in the absence of E2F1, there are no other proteins belonging to the E2F family, whose DNA binding activity is enhanced following the HMGA2/pRB interaction. Therefore, it is likely that other E2F-independent mechanisms are responsible for the pituitary alterations observed in the minority of these mice.

## Conclusion

Our data demonstrate that E2F1 activation is a crucial step required for the onset of pituitary adenomas in HMGA2 transgenic mice. Since HMGA2 amplification and overexpression has been detected also in human pituitary adenomas, we retain that E2F1 activation plays a critical role also in the human pituitary pathology.

These conclusions are not completely unexpected since several studies have previously demonstrated that alterations of the pRB/E2F pathway are critical for the development of pituitary adenomas in mice [45-47]. However, what appears to be really novel, is the mechanism that leads to E2F1 activation by HMGA2: the E2F1 protein is not displaced from the pRB complex, but an increased acetylation that is dependent on the removal of HDAC1 from pRB takes place. It would be very interesting to know whether the same mechanism may be induced by other proteins able to bind to the pRB complex and thereby are involved in pituitary tumorigenesis. To answer to this question, it would be interesting to evaluate the acetylation status of the E2F1 protein in pituitary adenomas when the HMGA2 is overexpressed or not. The presence of E2F1 hyperacetylation in the absence of HMGA2 overexpression would suggest the involvement of other proteins acting with the same or similar mechanism of HMGA2 protein, or alternatively other mechanisms that eventually lead to an increase in E2F1 acetylation and subsequent activation.
